# Inhibitory effect of nintedanib on VEGF secretion in retinal pigment epithelial cells induced by exposure to a necrotic cell lysate

**DOI:** 10.1371/journal.pone.0218632

**Published:** 2019-08-06

**Authors:** Makoto Hatano, Kazuhiro Tokuda, Yuka Kobayashi, Chiemi Yamashiro, Sho-Hei Uchi, Masaaki Kobayashi, Kazuhiro Kimura

**Affiliations:** Department of Ophthalmology, Yamaguchi University Graduate School of Medicine, Ube City, Yamaguchi, Japan; University of Florida, UNITED STATES

## Abstract

Necrosis is a form of cell death that results in rupture of the plasma membrane and the release of cellular contents, and it can give rise to sterile inflammation in the retina and other tissues. The secretion of vascular endothelial growth factor (VEGF) by retinal pigment epithelial (RPE) cells contributes to retinal homeostasis as well as to pathological angiogenesis. We have now examined the effect of a necrotic cell lysate prepared from human RPE cells (NLR) on the release of VEGF by healthy RPE cells. We found that NLR markedly increased the release of VEGF from RPE cells and that this effect was attenuated by nintedanib, a multiple receptor tyrosine kinase inhibitor, whereas it was unaffected by inhibitors of NF-κB signaling or of caspase-1. NLR also induced the phosphorylation of extracellular signal–regulated kinase (Erk) and signal transducer and activator of transcription 3 (Stat3) in a manner sensitive to inhibition by nintedanib, although inhibitors of Erk and Stat3 signaling pathways did not affect NLR-induced VEGF secretion. In addition, nintedanib attenuated the development of choroidal neovascularization in mice. Our results have thus shown that a necrotic lysate of RPE cells induced VEGF secretion from healthy RPE cells and that this effect was mediated by receptor tyrosine kinase signaling. They therefore suggest that VEGF secretion by healthy RPE cells is a potential therapeutic target for retinal diseases associated with sterile inflammation and pathological angiogenesis.

## Introduction

Inflammation is an initial response of organs or tissues to external or internal factors and contributes to homeostasis. The cellular contents released from damaged or necrotic cells can serve as a source of danger signals and play a role in the pathogenesis of various diseases associated with activation of the innate immune system [1, 2]. Pathogen-free inflammation induced by such cell damage or necrosis (sterile inflammation) is thus thought to contribute to several retinal diseases including diabetic retinopathy and age-related macular degeneration (AMD) [3, 4]. Sterile inflammation is associated with the release of cytokines and chemokines [5, 6] from various cell types in response to the activation of inflammasome-dependent or -independent signaling pathways including that mediated by nuclear factor (NF)–κB [7]. Focal adhesion–dependent signaling has also been implicated in sterile inflammation [8], as has signaling triggered by various nonimmune receptors including G protein–coupled receptors and receptor tyrosine kinases (RTKs) [9, 10].

The retinal pigment epithelium is the outermost layer of the retina, and retinal pigment epithelial (RPE) cells have many important functions such as the maintenance of photoreceptor excitability and formation of the blood-retinal barrier [11]. RPE cells also produce and release various growth factors that contribute to retinal homeostasis as well as to the response to pathological conditions including inflammation, necrosis, and apoptosis [12, 13]. An inflammatory response to damaged RPE cells is thought to be an initial event in drusen synthesis during the early phase of AMD [14]. Necrosis of RPE cells is a mediator of cell loss in AMD [4]. Medium conditioned by necrotic RPE cells has been shown to induce inflammatory gene expression in healthy RPE cells and in macrophages [15]. We have previously investigated the effects of endogenous danger signals on the release of pro-inflammatory cytokines and chemokines from RPE cells associated with sterile inflammation [16].

Vascular endothelial growth factor (VEGF) regulates development of the normal vasculature and contributes to tissue homeostasis [17]. It is produced by various cell types in response to external stimuli, with sterile inflammation having been shown to induce its secretion or expression in macrophages and endothelial cells [18]. In the eye, VEGF plays a role in physiological regulation of the retinal and choroidal vasculature [19]. It is also a key molecule in the induction of pathological angiogenesis associated with several retinal diseases including AMD, diabetic retinopathy, and retinopathy of prematurity [19]. Several ocular cell types including vascular endothelial cells, glial cells, macrophages, and RPE cells are able to produce and secrete VEGF [20]. VEGF expression has been shown to be regulated by extracellular signal–regulated kinase (Erk), Jak (Janus kinase)–Stat (signal transducer and activator of transcription), and PI3K (phosphoinositide 3-kinase)–Akt signaling pathways, all of which are activated by RTKs [21, 22].

Necrosis of RPE cells occurs as a result of inflammation during late phase of AMD [15, 16], but the detailed mechanism is controversial. We have now investigated the effect of a necrotic cell lysate prepared from human RPE cells on VEGF secretion from healthy RPE cells. We found that such a lysate indeed induced VEGF secretion from healthy RPE cells and that this effect was mediated by RTK signaling. We also show that the development of choroidal neovascularization (CNV) in vivo was attenuated by the RTK inhibitor nintedanib in a mouse model.

## Materials and methods

### Materials

Dulbecco’s modified Eagle’s medium–nutrient mixture F12 (DMEM-F12), penicillin, streptomycin, fetal bovine serum, and trypsin-EDTA were obtained from Invitrogen-Gibco (Rockville, MD), 24-well culture plates were from Corning (Corning, NY), and cell culture dishes were from Greiner Bio-One (Frickenhausen, Germany). A protease inhibitor cocktail was obtained from Sigma-Aldrich (St. Louis, MO). A Bio-Plex protein array system and Bio-Plex human cytokine assay were obtained from Bio-Rad (Hercules, CA). Rabbit polyclonal antibodies to human VEGF-A, biotinylated antibodies to human VEGF-A, and recombinant human VEGF-A for an enzyme-linked immunosorbent assay (ELISA) were obtained from R&D Systems (Minneapolis, MN). Rabbit polyclonal antibodies to total or phosphorylated forms of Erk1/2, to phosphorylated Stat3, or to total or phosphorylated forms of Akt were obtained from Cell Signaling (Beverly, MA), and mouse monoclonal antibodies to Stat3 were from Santa Cruz Biotechnology (Santa Cruz, CA). Mouse monoclonal antibodies to paxillin were obtained from BD Biosciences (Franklin Lakes, NJ), rabbit polyclonal antibodies to phosphorylated paxillin (Tyr^118^) were from Merck Millipore (Burlington, MA), and mouse monoclonal antibodies to β-actin were from Sigma-Aldrich. Horseradish peroxidase–conjugated horse antibodies to mouse immunoglobulin G (IgG) or goat antibodies to rabbit IgG were obtained from Cell Signaling, and an enhanced chemiluminescence (ECL) kit and nitrocellulose membranes were from Amersham Pharmacia Biotech (Uppsala, Sweden). An IκB kinase–2 (IKK-2) inhibitor was obtained from Calbiochem (La Jolla, CA), a caspase-1 inhibitor (VX-765) and Erk signaling inhibitor (PD98059) were from InvivoGen (San Diego, CA), a Jak-Stat3 signaling inhibitor (AG490) was from R&D Systems, and an RTK inhibitor (nintedanib) was from Chem Scene (Monmouth Junction, NJ). Ketamine hydrochloride and xylazine were obtained from Daiichi Sankyo (Tokyo, Japan) and Bayer (Leverkusen, Germany), respectively. Rabbit monoclonal antibodies to VEGF-A for immunofluorescence analysis were obtained from Abcam (Cambridge, UK), and Alexa Fluor 488–conjugated isolectin B4 and Alexa Fluor 568–conjugated secondary antibodies were from Invitrogen (Carlsbad, CA).

### RPE cell culture

The human RPE cell line ARPE-19 was obtained from American Type Culture Collection (Manassas, VA) and was cultured in DMEM-F12 supplemented with 10% fetal bovine serum, penicillin (100 U/mL), and streptomycin (100 μg/mL) [23]. The cells were maintained in 100-mm cell culture dishes at 37°C under a humidified atmosphere of 5% CO_2_, and they were passaged every 5 to 7 days. They were detached from the dishes after achieving confluence by exposure to trypsin-EDTA and were diluted 1:3 or 1:4. Cells between passages 20 and 25 were used for experiments.

### Preparation of a necrotic cell lysate

A necrotic cell lysate of ARPE-19 cells (NLR) was prepared as described previously [16, 24]. In brief, ARPE-19 cells were washed once with phosphate-buffered saline (PBS) and exposed to trypsin-EDTA for 3 min, after which the detached cells were collected in DMEM-F12, washed twice with PBS, resuspended at a density of 1 × 10^6^ cells/mL in DMEM-F12, and subjected to three rounds of rapid freezing in liquid nitrogen and thawing in a water bath at 37°C. The cell suspension was then centrifuged at 20,000 × *g* for 10 min at 4°C, and the resulting supernatant was collected as NLR.

### Assay of VEGF secretion

ARPE-19 cells were grown to 100% confluence in 24-well plates, cultured in DMEM-F12 alone for 24 h, and then stimulated with 500 μL of NLR for an additional 24 h. For evaluation of the effects of inhibitors on NLR-induced VEGF release, serum-deprived ARPE-19 cells were incubated for 1 h in the absence or presence of inhibitor and then for 24 h in the additional presence of NLR. The culture supernatants were collected, centrifuged at 20,000 × *g* for 5 min at 4°C to remove debris, and frozen at –80°C until subsequent measurement of VEGF-A concentration with the use of a multiplex human cytokine assay system or an ELISA.

### Immunoblot analysis

ARPE-19 cells were grown to 100% confluence in 60-mm cell culture dishes and then cultured in DMEM-F12 alone for 24 h. For evaluation of the effects of nintedanib on NLR-induced paxillin expression or phosphorylation of paxillin, Erk1/2, Stat3, and Akt, the serum-deprived cells were incubated for 1 h in the absence or presence of nintedanib and then for 24 h in the additional presence of NLR. For immunoblot analysis, the cells were washed twice with PBS and then lysed in 300 μL of a solution containing 1% Nonidet P-40, 50 mM Tris-HCl (pH 7.4), 100 mM NaCl, 10 mM MgCl_2_, 1 mM dithiothreitol, 1 mM phenylmethylsulfonyl fluoride, and 1% protease inhibitor cocktail. The cell lysates were homogenized and then centrifuged at 20,000 × *g* for 5 min at 4°C, and the resulting supernatants were stored at –80°C until analysis. The cell lysates (20 μg of protein) were subsequently fractionated by SDS-polyacrylamide gel electrophoresis on a 10% gel, the separated proteins were transferred electrophoretically to a nitrocellulose membrane, and nonspecific sites of the membrane were then blocked before incubation with primary antibodies (each at a 1:1000 dilution). Immune complexes were detected with secondary antibodies and ECL reagents.

### Laser-induced CNV in mice

Animal experiments were approved by the animal ethics committee of Yamaguchi University Graduate School of Medicine. Female 8-week-old C57BL/6 mice (*n* = 32) were obtained from Japan SLC (Shizuoka, Japan). Mice were anesthetized by intraperitoneal injection of ketamine (90 mg/kg) and xylazine (10 mg/kg), and the right eye was subjected to laser photocoagulation with settings including a light wavelength of 532 nm, spot size of 75 μm, pulse duration of 0.1 s, and laser power of 200 mW. The mice were then randomized to two groups of 16 and treated by injection of PBS (2 μl) containing either 100 μM nintedanib or dimethyl sulfoxide vehicle into the vitreous cavity. After 7 days, the animals were killed by cervical dislocation, and the right eye was enucleated and fixed in 4% paraformaldehyde for 1 h on ice. Choroidal flat-mount preparations were washed with PBS, exposed to 100% methanol at room temperature for 10 min, and incubated first for 1 h at room temperature with 5% dried skim milk in PBS and then for 24 h at 4°C with Alexa Fluor 488–conjugated isolectin B4 (for evaluation of CNV) and with antibodies to VEGF, each diluted 1:100 in PBS. VEGF immune complexes were detected by additional incubation for 90 min at room temperature with Alexa Fluor 568–conjugated secondary antibodies (1:1000 dilution). The preparations were then mounted with 50% glycerol in PBS and examined with a BZ-X710 fluorescence microscope (Keyence). The area of CNV was measured with the use of ImageJ software.

### Statistical analysis

Quantitative data are presented as means + SD. The multiplex human cytokine assay and immunoblot analysis were performed in triplicate, whereas the ELISA was performed in quadruplicate, and all experiments were repeated at least three times. Statistical analysis was performed with Dunnett’s multiple comparison test or Student’s *t* test. A *P* value of <0.05 was considered statistically significant.

## Results

### Effect of NLR on VEGF secretion from RPE cells

**Fig 1:**
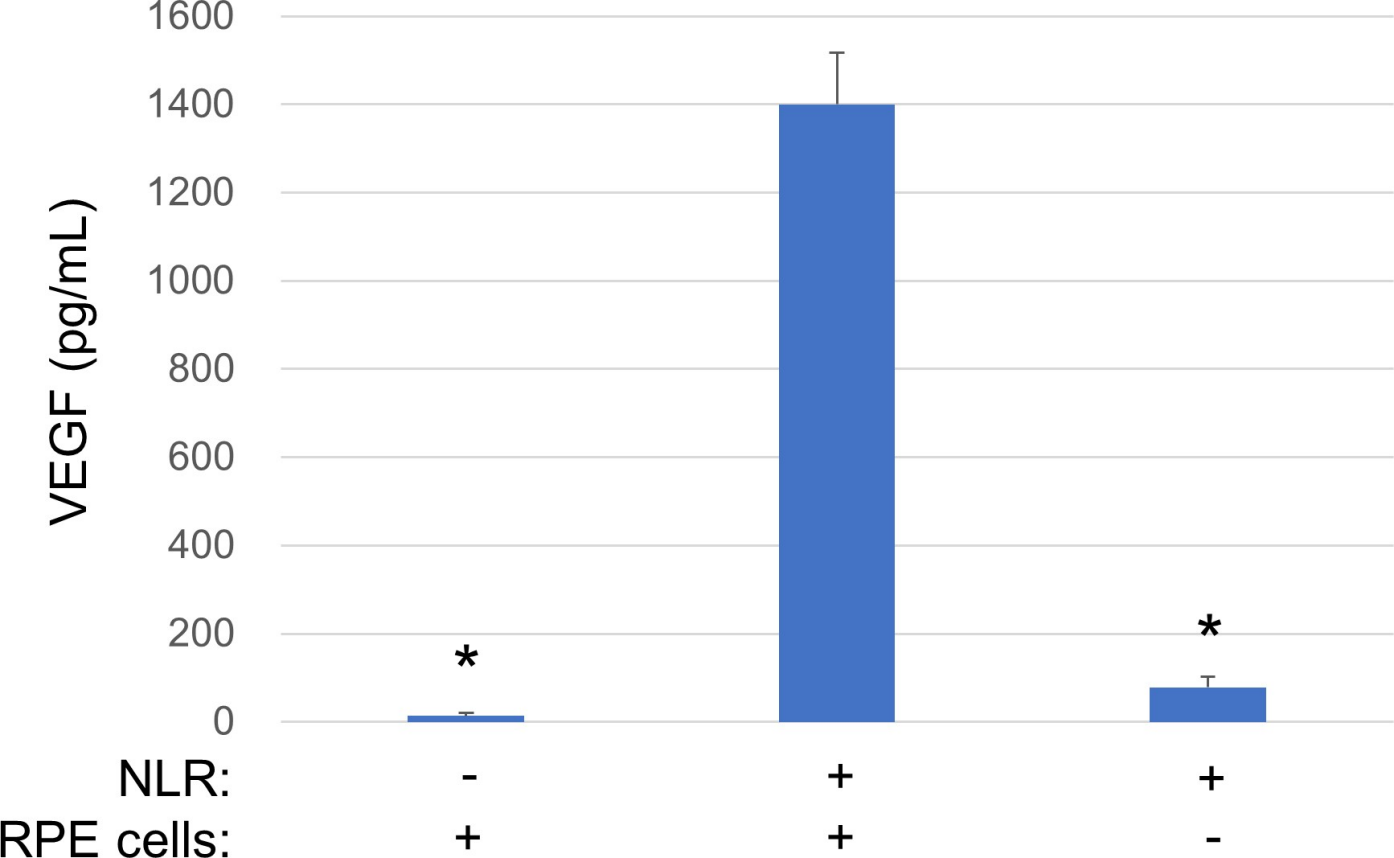
Effect of NLR on the secretion of VEGF from RPE cells. Serum-deprived cells were incubated for 24 h in serum-free medium or with NLR, after which culture supernatants were collected for measurement of VEGF with a multiplex assay system. The concentration of VEGF in NLR alone was measured as a control. Data are means + SD from four independent experiments. *P < 0.05 versus cells incubated with NLR (Dunnett’s test).

We first examined whether a necrotic cell lysate derived from human RPE cells (NLR) is able to stimulate VEGF secretion from healthy RPE cells. The healthy cells were incubated in serum-free medium for 24 h before exposure to NLR for 24 h, after which the culture supernatants were collected for analysis with a multiplex assay system. The concentration of VEGF in culture supernatants of cells exposed to NLR was markedly increased compared with that either in culture supernatants of cells incubated without NLR or in NLR itself (Fig 1).

### RTK signaling is required for NLR-induced VEGF secretion from RPE cells

**Fig 2:**
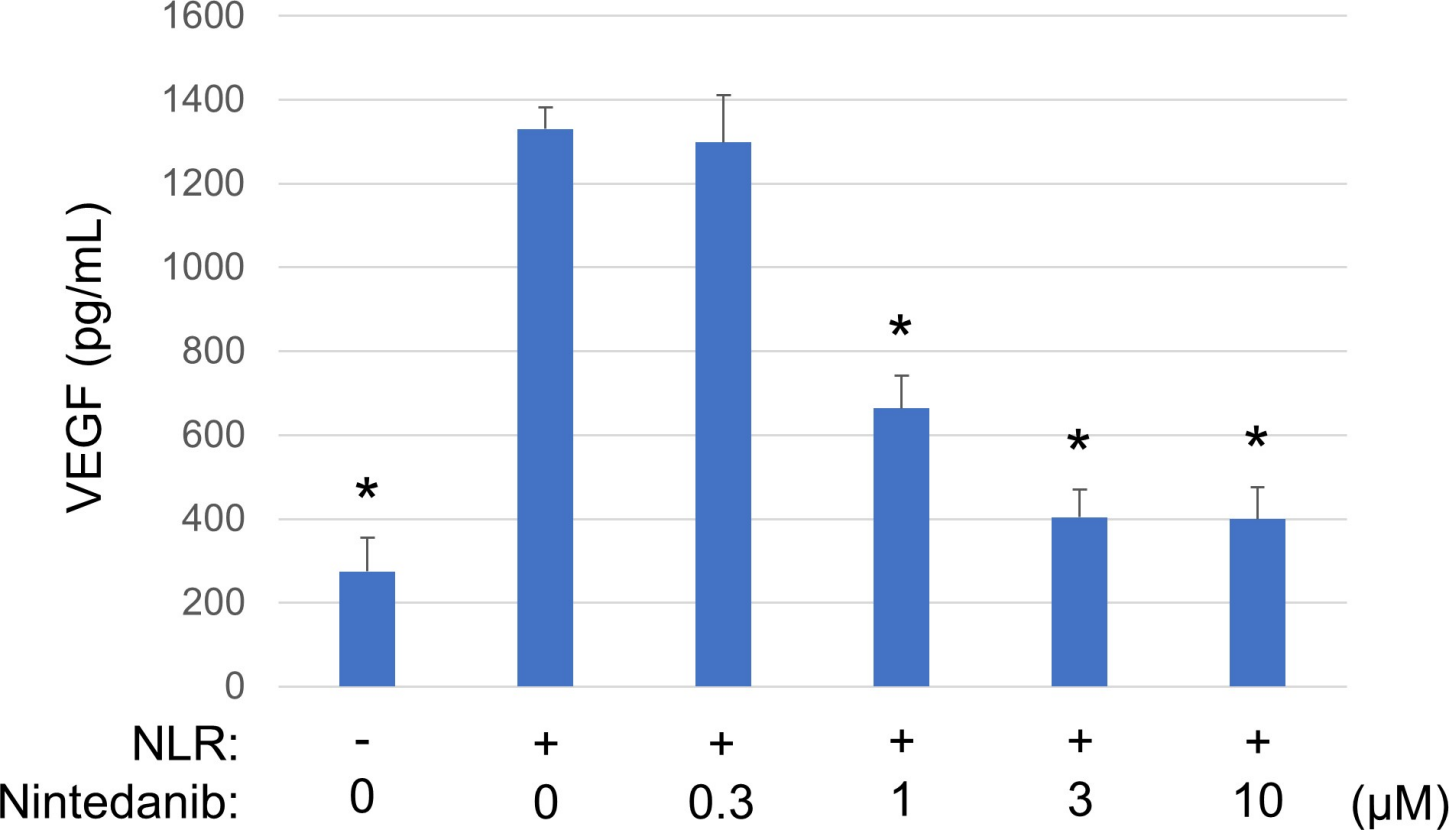
Effect of nintedanib on NLR-induced VEGF secretion from RPE cells. Serum-deprived cells were incubated first for 1 h with various concentrations of nintedanib and then for 24 h in the additional absence or presence of NLR, after which culture supernatants were collected for measurement of VEGF with an ELISA. Data are means + SD from four independent experiments. *P < 0.05 versus cells incubated with NLR but not exposed to nintedanib (Dunnett’s test).

VEGF expression has been shown to be regulated through activation of RTKs [21, 22]. To investigate whether RTK signaling might contribute to NLR-induced VEGF secretion from RPE cells, we examined the effect of a multiple RTK inhibitor (nintedanib). The cells were exposed to nintedanib (0.3 to 10 μM) for 1 h before incubation for 24 h in the additional presence of NLR. The NLR-induced secretion of VEGF from RPE cells as measured with an ELISA was inhibited by nintedanib in a concentration-dependent manner (Fig 2).

### Inflammasome-dependent or -independent pathways of sterile inflammation are not required for NLR-induced VEGF secretion from RPE cells

**Fig 3:**
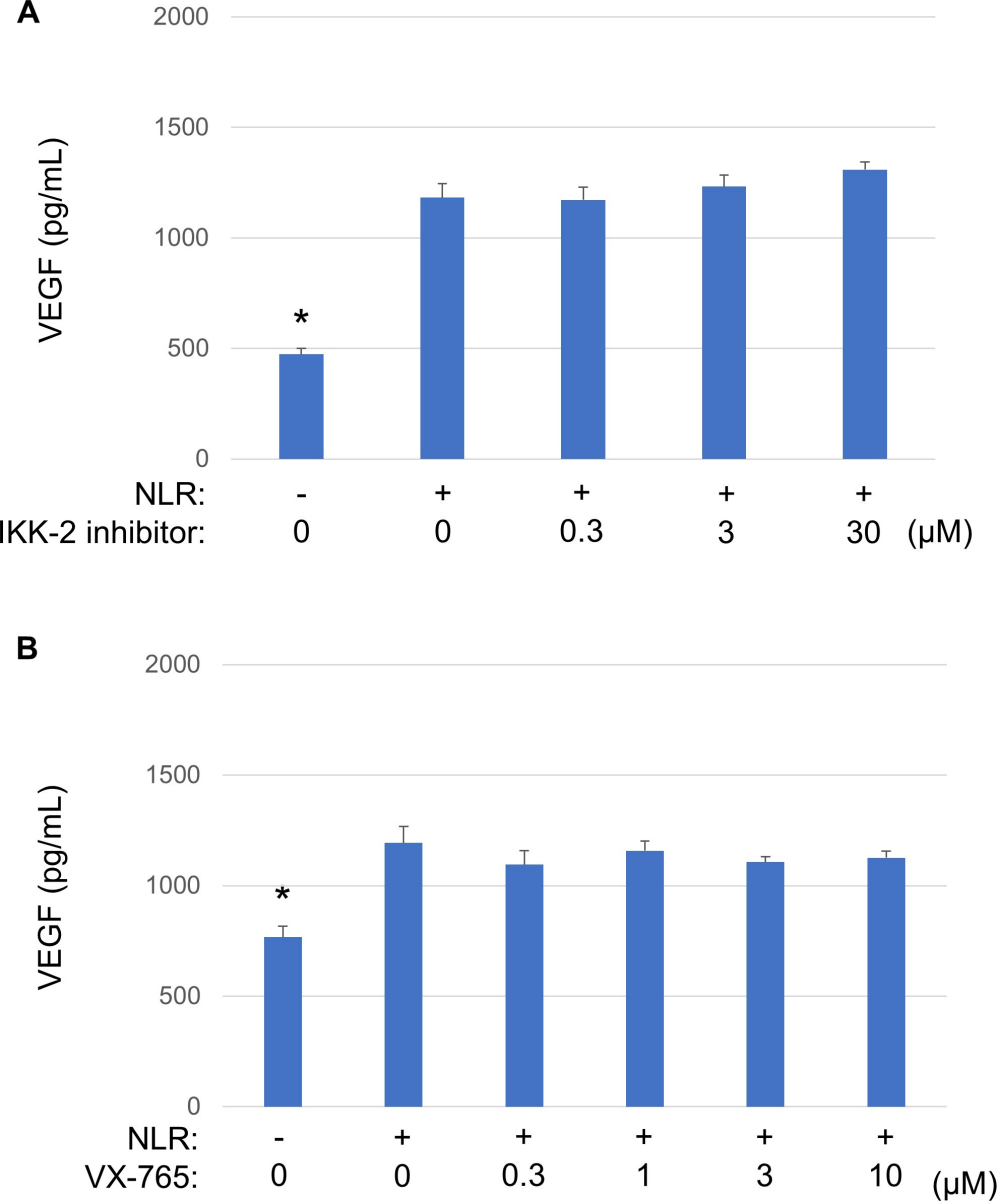
Effects of an IKK-2 inhibitor and a caspase-1 inhibitor (VX-765) on NLR-induced VEGF secretion from RPE cells. Serum-deprived cells were incubated first for 1 h with various concentrations of an IKK-2 inhibitor (**A**) or VX-765 (**B**) and then for 24 h in the additional absence or presence of NLR, after which culture supernatants were collected for measurement of VEGF with an ELISA. Data are means + SD from four independent experiments. *P < 0.05 versus cells incubated with NLR but not exposed to inhibitor (Dunnett’s test).

Inflammasome-dependent and -independent signaling pathways contribute to sterile inflammation [7, 25]. To investigate whether such signaling pathways might play a role in NLR-induced VEGF secretion from RPE cells, we examined the effects of an IKK-2 inhibitor, which blocks NF-κB signaling, and of a caspase-1 inhibitor (VX-765). The cells were exposed to the IKK-2 inhibitor (0.3 to 30 μM) or VX-765 (0.3 to 10 μM) for 1 h before incubation for 24 h in the additional presence of NLR and measurement of VEGF secretion with an ELISA. The NLR-induced secretion of VEGF from RPE cells was not affected by either the IKK-2 inhibitor or VX-765 (Fig 3).

### Effect of NLR on focal adhesion signaling in RPE cells

**Fig 4:**
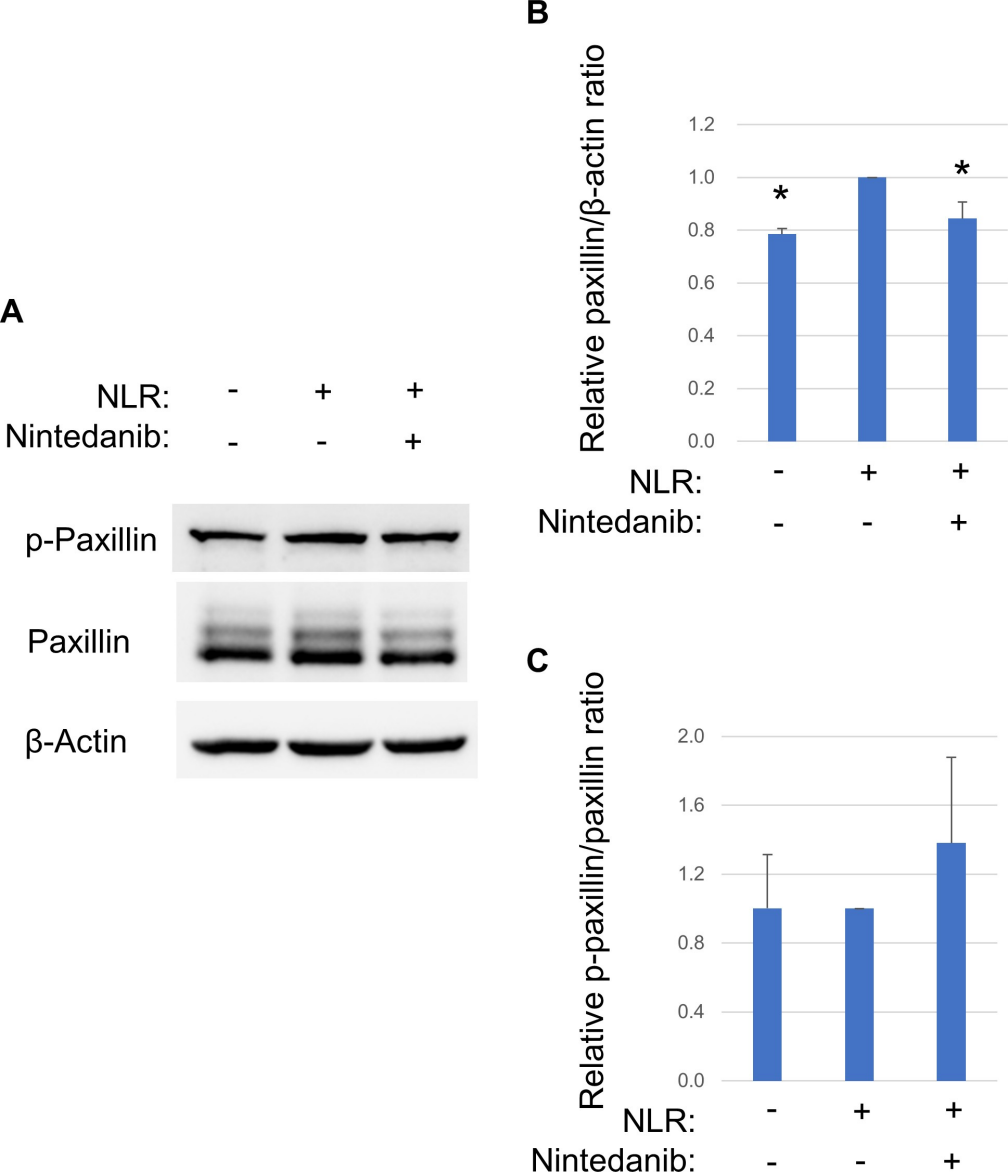
Effects of NLR and nintedanib on the expression and phosphorylation of paxillin in RPE cells. (**A**) Serum-deprived cells were exposed to nintedanib (10 μM) for 1 h before incubation for 24 h in the additional absence or presence of NLR, after which cell lysates were collected for immunoblot analysis with antibodies to total or phosphorylated (p-) forms of paxillin and to β-actin (loading control). (**B**, **C**) Blots similar to that in (A) were subjected to densitometry to determine paxillin band intensity normalized by β-actin band intensity (**B**) as well as phosphorylated paxillin band intensity normalized by paxillin band intensity (**C**). Data are expressed as relative values and are means + SD from three independent experiments. *P < 0.05 versus cells incubated with NLR but not exposed to nintedanib (Dunnett’s test).

The focal adhesion signaling pathway has also been implicated in sterile inflammation [8]. We therefore examined whether NLR and nintedanib might affect the expression or phosphorylation of the focal adhesion–associated protein paxillin in RPE cells. The cells were exposed to nintedanib (10 μM) for 1 h before incubation for 24 h in the additional presence of NLR. Immunoblot analysis revealed that NLR increased the expression of paxillin and that this effect was attenuated by nintedanib (Fig 4A and 4B). The tyrosine phosphorylation of paxillin, however, was not affected by NLR or nintedanib (Fig 4A and 4C).

### Effects of NLR on Erk, Stat3, and Akt signaling in RPE cells

**Fig 5:**
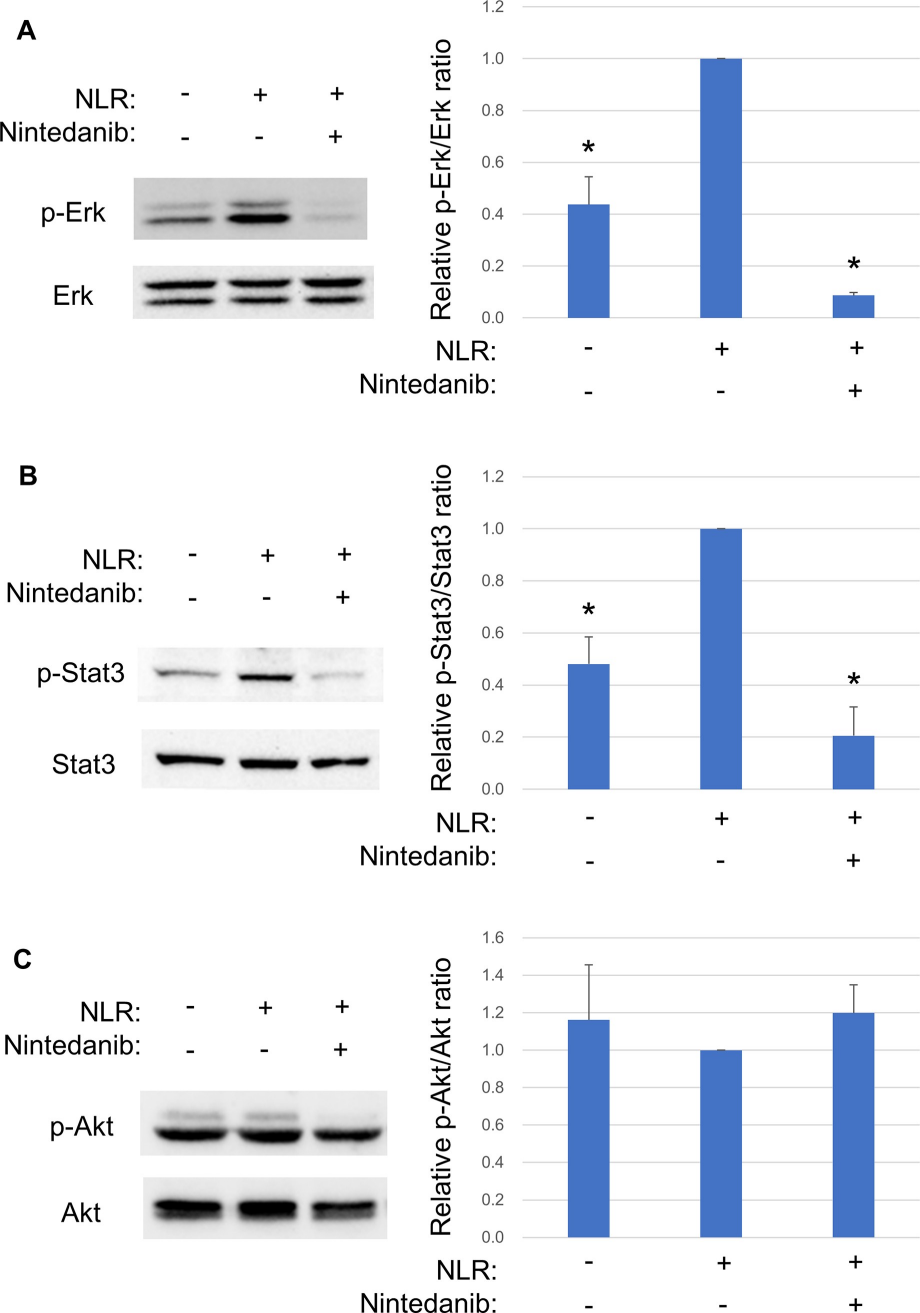
Effects of NLR and nintedanib on Erk1/2, Stat3, and Akt phosphorylation in RPE cells. Serum-deprived cells were exposed to nintedanib (10 μM) for 1 h before incubation for 24 h in the additional absence or presence of NLR, after which cell lysates were collected for immunoblot analysis of total and phosphorylated (p-) forms of Erk1/2 (**A**), Stat3 (**B**), and Akt (**C**). Representative blots are shown together with quantitative data for the band intensity of each phosphorylated protein normalized by that of the corresponding total protein from three independent experiments. The data are expressed as relative values and are means + SD. *P < 0.05 versus cells incubated with NLR but not exposed to nintedanib (Dunnett’s test).

VEGF expression is regulated by Erk, Jak-Stat, and PI3K-Akt signaling pathways [21, 22]. We therefore examined whether NLR and nintedanib might affect the phosphorylation of Erk1/2, Stat3, or Akt in RPE cells. The cells were exposed to nintedanib (10 μM) for 1 h before incubation for 24 h in the additional presence of NLR. Immunoblot analysis revealed that NLR induced the phosphorylation of Erk1/2 and Stat3 in a manner sensitive to inhibition by nintedanib (Fig 5A and 5B). NLR had no effect on the phosphorylation of Akt (Fig 5C).

### Effects of Erk and Jak-Stat3 signaling inhibitors on NLR-induced VEGF secretion from RPE cells

**Fig 6:**
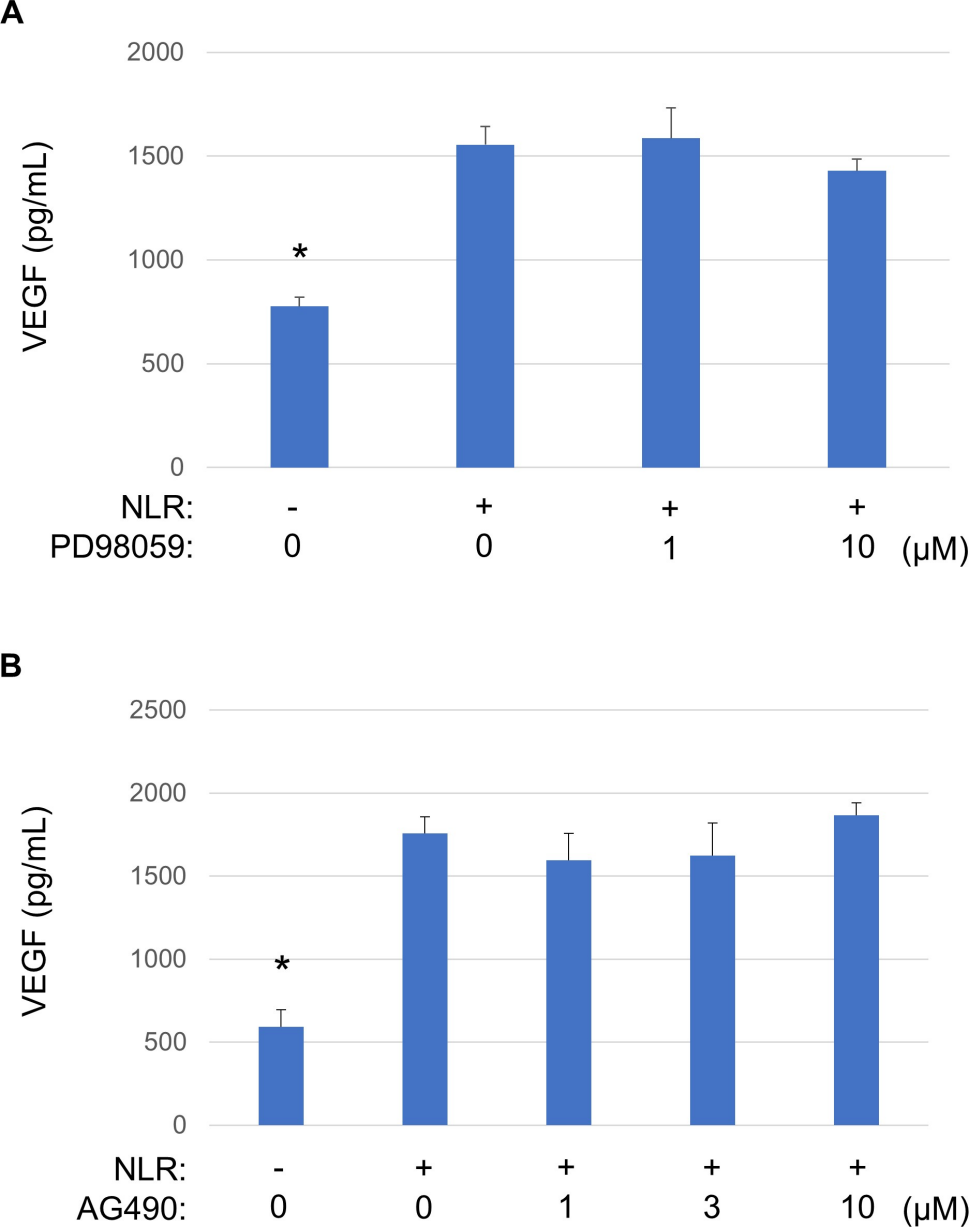
Effects of Mek-Erk (PD98059) and Jak-Stat3 (AG490) signaling inhibitors on NLR-induced VEGF secretion from RPE cells. Serum-deprived cells were incubated first for 1 h with various concentrations of PD98059 (**A**) or AG490 (**B**) and then for 24 h in the additional absence or presence of NLR, after which culture supernatants were collected for measurement of VEGF with an ELISA. Data are means + SD from four independent experiments. *P < 0.05 versus cells incubated with NLR but not exposed to inhibitor (Dunnett’s test).

Given that NLR induced the phosphorylation of Erk1/2 and Stat3 in RPE cells (Fig 5A and 5B), we examined the effects of inhibitors of such phosphorylation on NLR-induced VEGF secretion. The cells were thus exposed to the Erk kinase (Mek) inhibitor PD98059 (1 or 10 μM) or the Jak inhibitor AG490 (1 to 10 μM) for 1 h before incubation for 24 h in the additional presence of NLR and measurement of VEGF secretion with an ELISA. The NLR-induced secretion of VEGF from RPE cells was not affected by either PD98059 or AG490 (Fig 6).

### Effect of nintedanib on CNV in mice

**Fig 7:**
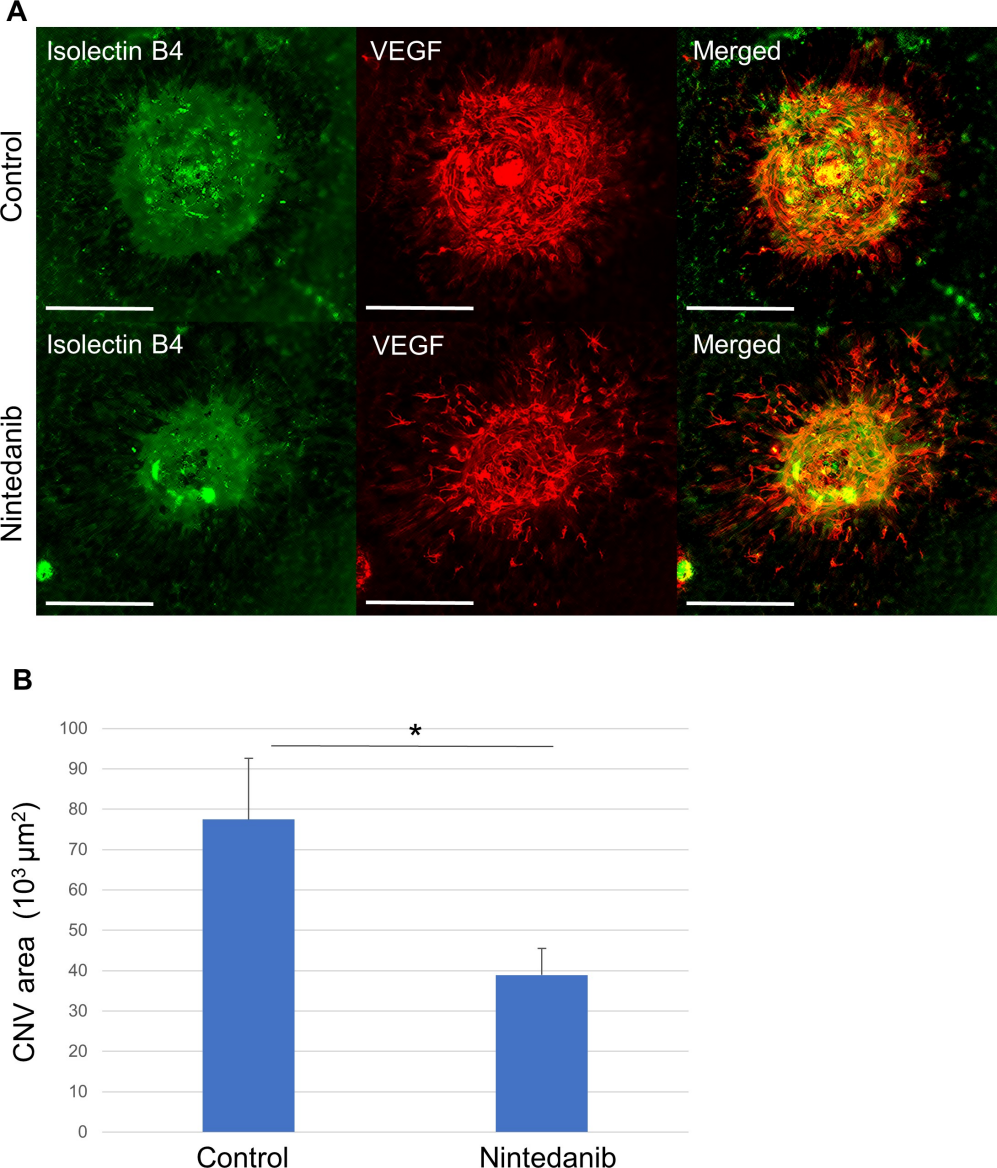
Effect of nintedanib on CNV development in mice. (**A**) Choroidal flat-mount preparations from mice at 7 days after photocoagulation and intravitreous injection of 100 μM nintedanib or vehicle (control) were subjected to fluorescence staining with isolectin B4 and antibodies to VEGF. Scale bars, 200 μm. (**B**) The area of CNV in mice treated as in (A) was determined. Data are means + SD for 16 eyes per condition. *P < 0.05 (Student’s *t* test).

We examined the effect of nintedanib on the development of CNV in a mouse model in which the choroid, Bruch’s membrane, and RPE cells are disrupted or destroyed [26] and which mimics the pathological processes of AMD [27]. Fluorescence microscopic analysis of choroidal flat-mount preparations stained with isolectin B4 showed that injection of nintedanib into the vitreous cavity after photocoagulation significantly attenuated the extent of CNV apparent at 7 days (Fig 7). Expression of VEGF was confirmed in an area largely overlapping with that of CNV under both control and nintedanib conditions, but it was too discontinuous for area measurement (Fig 7A).

### Discussion

RPE cells perform many important functions, among which we focused on the production of VEGF. Pathological secretion of VEGF can induce not only angiogenesis but also inflammation in the eye [28, 29]. To examine the pathological activation of RPE cells in sterile inflammation, which is associated with several retinal diseases including AMD and diabetic retinopathy [3, 4], we investigated the effects of a necrotic cell lysate derived from RPE cells on VEGF secretion from healthy RPE cells. We found that NLR markedly induced the secretion of VEGF from RPE cells, suggesting that healthy RPE cells up-regulate VEGF production in response to the injury or death of neighboring RPE cells. This response may thus contribute to pathological VEGF expression in retinal diseases such as diabetic retinopathy and AMD.

VEGF expression is regulated by the activation of RTKs during angiogenesis and leukemogenesis [21, 22]. We therefore examined the effect of nintedanib, a multiple RTK inhibitor, on NLR-induced VEGF secretion from RPE cells. Nintedanib binds to the intracellular tyrosine kinase domain of VEGF receptors 1 to 3, fibroblast growth factor receptors 1 to 3, and platelet-derived growth factor receptors α and β [30, 31]. We found that nintedanib inhibited the NLR-induced secretion of VEGF from RPE cells in a concentration-dependent manner, consistent with the notion that this effect of NLR is mediated by RTK signaling. VEGF expression has also been shown to be regulated by Erk, Jak-Stat, and PI3K-Akt signaling pathways [21, 22]. We found that NLR induced the phosphorylation of Erk1/2 and Stat3 in RPE cells and that these effects were inhibited by nintedanib. The NLR-induced secretion of VEGF was not attenuated by inhibitors of Mek-Erk or Jak-Stat3 signaling, however, suggesting that, although these signaling pathways are activated downstream of RTK activation by NLR, they do not mediate the effect of NLR on VEGF secretion.

Two key signaling pathways mediate sterile inflammation. In the inflammasome-independent signaling pathway, inflammation is triggered by the activation of NF-κB [25]. We previously showed that NLR induced the phosphorylation and degradation of the endogenous NF-κB inhibitor IκB-α and thereby triggered the nuclear translocation of NF-κB in RPE cells [16]. However, we here found that NLR-induced VEGF secretion from RPE cells was not attenuated by an IKK-2 inhibitor that blocks NF-κB signaling. The activation of caspase-1 plays an important role in the inflammasome-dependent signaling pathway [7, 25]. However, we again found that NLR-induced VEGF secretion from RPE cells was not affected by a caspase-1 inhibitor. These results thus suggest that the up-regulation of VEGF secretion from RPE cells during sterile inflammation is not mediated by the canonical inflammasome-dependent or -independent signaling pathways.

Focal adhesion signaling has also been implicated in sterile inflammation [8], in RTK signaling [32], and in VEGF secretion from RPE cells [33, 34]. Tyrosine phosphorylation of paxillin [35] and an increase in paxillin expression [36] have been thought to reflect activation of focal adhesions in RPE cells. We here found that NLR had no effect on the tyrosine phosphorylation of paxillin in RPE cells. Given that serine-threonine phosphorylation of paxillin also contributes to focal adhesion signaling [37], it remains possible that NLR may induce such phosphorylation of paxillin. We did find that NLR increased the expression of paxillin in RPE cells and that this effect was attenuated by nintedanib, suggesting that focal adhesion signaling is triggered downstream of RTK activation and may contribute to the up-regulation of VEGF secretion.

VEGF contributes to the induction of CNV [38, 39]. Laser-induced CNV is a model of injury and inflammation-induced angiogenesis associated with AMD [27, 40]. In this model, RPE cells are directly damaged by laser irradiation [26]. RTK signaling [41, 42] as well as Erk, Jak-Stat3, and PI3K-Akt pathways [43, 44] are implicated in the laser-induced CNV model in mice. We found that nintedanib suppressed the development of neovascularization and VEGF expression in the laser-induced CNV model. The up-regulation of VEGF expression by RTK signaling in RPE cells thus likely contributes to CNV development in this model. In addition to RPE cells, other cell types including macrophages, endothelial cells, and glial cells might up-regulate VEGF expression in response to photocoagulation [45–47]. VEGF receptors are among the RTKs targeted by nintedanib, and their inhibition may therefore also contribute to the effect of nintedanib on CNV in this model. The role of VEGF expression in healthy RPE cells induced by necrosis of neighboring RPE cells in CNV induction thus requires further evaluation.

We previously analyzed NLR for various cytokines and detected tumor necrosis factor–α as well as interleukin (IL)–1α and IL-1β [16]. NLR thus likely contains several endogenous danger signals. Such danger signals were previously shown to induce VEGF gene transcription in dendritic cells [48]. Further studies will be required to identify the component (or components) of NLR that are responsible for the induction of VEGF secretion from RPE cells. We prepared NLR by subjecting RPE cells to freezing and thawing. Given that RPE cell necrosis is also induced by oxidative stress such as that triggered by hydrogen peroxide or *tert*-butyl hydroperoxide [15], it will be of interest to determine whether necrotic extracts prepared from such stressed RPE cells also induce the secretion of VEGF from healthy RPE cells.

Laser irradiation destroys or induces necrosis in retinal cells including RPE cells [49] and therefore likely results in the exposure of remaining intact RPE cells to the contents of necrotic cells. Several cytokines such as IL-6, IL-8, and monocyte chemoattractant protein–1 have been implicated in CNV [50, 51], and we previously showed that NLR contains these cytokines [16]. Growth factors such as fibroblast growth factor and platelet-derived growth factor in addition to VEGF are also thought to play a role in CNV development [52]. It is therefore likely that the effects of laser irradiation on CNV are mediated at least in part by the contents of necrotic RPE cells. It will thus be of interest to determine the effect of intravitreal injection of NLR on CNV and to compare such an effect with that of laser irradiation.

Although we detected VEGF expression by immunostaining in an area largely overlapping with that of CNV under both control and nintedanib conditions, the staining was too discontinuous for area measurement. It will therefore be of interest to examine VEGF expression by other approaches such as in situ hybridization. In addition, CNV can be detected by immunostaining for intercellular adhesion molecule (ICAM)–2 [53] as well as by isolectin B4 staining. We detected CNV with isolectin B4 in the present study, but examination of the effect of nintedanib on laser-induced CNV by immunostaining of ICAM-2 would also be of interest.

In conclusion, we have shown that a necrotic cell lysate prepared from RPE cells stimulated the secretion of VEGF from healthy RPE cells and that this effect was mediated by RTK signaling, with downstream focal adhesion signaling possibly also playing a role. Targeting of the pathological secretion of VEGF from RPE cells with a multi-RTK inhibitor may thus be a potential therapeutic approach to the resolution of sterile inflammation and angiogenesis associated with retinal diseases.

## Supporting information

S1 Fig A summary of minimal data set. Values, Mean, S.D., Statistics method used and P value for each Figs 1–7 were summarized.
(XLSX)
